# Microstructural Characterization, Porosity Anisotropy, and Residual Stress Fields in ADAM-Fabricated 17-4PH Stainless Steel

**DOI:** 10.3390/ma19173684

**Published:** 2026-08-30

**Authors:** Peter Spuro, Andrej Czan, Michal Sajgalik, Mario Drbúl, Marek Roszak, Oktawian Bialas, Marek Sadilek, Abdesselam Mechali

**Affiliations:** 1 Department of Machining and Manufacturing Technology, Faculty of Mechanical Engineering, University of Žilina, Univerzitná 1, 010 26 Žilina, Slovakia; andrej.czan@fstroj.uniza.sk (A.C.); michal.sajgalik@fstroj.uniza.sk (M.S.); mario.drbul@fstroj.uniza.sk (M.D.); 2 Department of Engineering Materials and Biomaterials, Faculty of Mechanical Engineering, Silesian University of Technology, Konarskiego 18A, 44-100 Gliwice, Poland; marek.roszak@polsl.pl (M.R.); oktawian.bialas@polsl.pl (O.B.); 3 Department of Machining, Assembly and Engineering Metrology, Faculty of Mechanical Engineering, VSB—Technical University of Ostrava, 17. listopadu 2172/15, 708 00 Ostrava-Poruba, Czech Republic; marek.sadilek@vsb.cz (M.S.); abdesselam.mechali.st@vsb.cz (A.M.)

**Keywords:** Atomic Diffusion Additive Manufacturing (ADAM), 17-4PH stainless steel, porosity anisotropy, residual stress

## Abstract

This work presents an experimental characterization of 17-4PH stainless steel fabricated by Atomic Diffusion Additive Manufacturing (ADAM). The microstructure, porosity, local chemical composition, and residual stresses were investigated using optical microscopy, SEM-EDS, digital image analysis, and sin^2^ψ X-ray diffraction. Pronounced porosity anisotropy was observed, with a lower porosity area fraction in the transverse section (1.45%) than in the longitudinal section (3.48%), where elongated channel-like inter-layer defects were identified. Isothermal sintering at 1315 °C produced a predominantly martensitic microstructure with equiaxed morphology. Local chemical variations were detected in selected macro-voids and interfacial regions, including elevated concentrations of C, Cr, and Nb, reaching 1.61 wt.%, 42.58 wt.%, and 16.31 wt.%, respectively. These anomalies may be related to localized binder-derived residues or secondary phase formation, although their origin cannot be conclusively determined by EDS alone. Residual stress measurements at 12 surface locations revealed spatial variations, with maximum axial tensile stress of 185.6 ± 21.9 MPa and local compressive stress of −46.7 ± 10.9 MPa. All measured stresses remained below the reported yield strength. The findings highlight the importance of optimizing inter-layer bonding and thermal debinding conditions in ADAM.

## 1. Introduction

Metal additive manufacturing (AM) has become a strategic process for the production of complex industrial components with a high degree of geometrical freedom and functional integration [1]. The use of machine learning for materials development [2] and the need to overcome technological barriers for wider market up-take [3] are propelling continuous scientific progress in this field. Detailed knowledge of metallurgy and processing science is needed for successful fabrication of AM processes, according to a comprehensive review of AM processes [4,5]. AM is becoming a key technology for the production of complex shaped parts [6,7]. This is even more true in the aerospace sector where the interdependence between process parameters, microstructure and resulting properties, is what defines the integrity of the whole system [8]. A key aspect of the process is the formation of residual stresses from non-uniform thermal loading in metal additive manufacturing [9]. These internal stresses coupled with high thermal gradients can have a fundamental effect on the dimensional stability and mechanical integrity of the produced parts [10]. In processes which require high energy input, these stresses can cause microcracking and other structural discontinuities [11]. Residual stresses, which promote crack initiation, consequently limit the cyclic endurance of the material regarding its fatigue performance [12]. The comparison of costs and industrial applications of AM technologies indicates the need for effective control of those phenomena for safe operation of components [13]. The final density and reinforcement mechanisms of the material are determined by the thermal behavior and densification mechanisms [14]. The accuracy of laser-based methods is high; however there are still problems with process instabilities and spatter generation [15,16]. Binder jetting likewise requires careful control of powder characteristics, green-part printing, debinding, and sintering to achieve sufficiently dense parts [17,18]. Furthermore, the research is strictly focused on the surface quality and the control of the precursor powder in order to optimize the scanning strategies and reduce the defect rate [19,20,21,22,23]. The central part of this work is the Atomic Diffusion Additive Manufacturing (ADAM) process, where filament extrusion is followed by debinding and sintering. The mechanical behavior of the 17-4PH stainless steel produced by this process presents some particularities according to the metallurgical processing conditions [24]. The analysis of the density and dimensional accuracy of the ADAM process shows the capability of this technology to compete with traditional powder metallurgy methods [25,26]. ISO metrology standards [27,28] for geometrical product specification and technical documentation are required to assure quality. The material integrity can be further modified by post-treatment processes such as Hot Isostatic Pressing (HIP) [29,30]. The optimization of stainless steel parts quality is a crucial process where powder characterization, feedstock monitoring and dimensional accuracy are key throughout the production cycle [31,32,33]. Recent works in the field of the metal material extrusion (MEX) for 17-4PH steel are focused on the mechanical and metrological characterization of the structures [34]. Strict standards of areal surface texture and X-ray diffraction analysis are defined for the assessment of surface quality and residual stresses [35,36]. Pore formation in AM processes is a complex multi-physics phenomenon, which requires accurate modeling of the extrusion tracks [37,38,39]. The fatigue behavior of additively manufactured 17-4PH steel is greatly affected by the interaction between surface roughness and heat treatment, which can eliminate the negative effects of residual stresses [40,41]. Post-processing microstructure and corrosion resistance confirm the need to control internal stresses for long-term stability [42,43,44]. Finally, the influence of surface finish on the mechanical properties of 17-4PH steel manufactured with the ADAM process is an important aspect for the final certification of the components [45]. While traditional solid-state sintering methods like Metal Injection Molding (MIM) or Binder Jetting (BJ) rely on capillary wicking, solvent debinding, or thin-wall component designs to remove binders, Atomic Diffusion Additive Manufacturing (ADAM) utilizes thick, highly loaded multi-component polymer backbones extruded layer by layer. This specific feedstock architecture significantly alters the thermal debinding kinetics; the dense filament cross-sections restrict gas diffusion pathways for volatile degradation products. Consequently, incomplete polymer depolymerization poses a higher localized risk of residual carbonaceous retention, leading to potential carbonization and secondary carbide precipitation along prior particle boundaries during subsequent high-temperature sintering [46,47]. The present work focuses on the experimental characterization of 17-4PH stainless steel fabricated by the ADAM process, with particular emphasis on porosity anisotropy, microstructural features, local chemical heterogeneity, and residual stress distribution.

## 2. Materials and Methods

The 17-4 PH (AISI 630) stainless steel feedstock used for the Atomic Diffusion Additive Manufacturing (ADAM) process was supplied by Markforged Inc. (Waltham, MA, USA) in the form of a composite filament consisting of metal powder encapsulated in a thermoplastic binder. The chemical composition and specification limits presented in Table 1 were obtained from the manufacturer’s technical datasheet for Markforged 17-4 PH stainless steel [48]. These values represent the manufacturer’s declared chemical composition specifications for the metallic material and were not independently determined by chemical analysis of the filament used in the present study.

### 2.1. Specimen Fabrication and Printing Parameters

The test specimens were produced using the Metal X 3D printing system (Markforged Inc., Waltham, MA, USA) based on Atomic Diffusion Additive Manufacturing (ADAM), an indirect additive manufacturing process which uses a composite filament made of 17-4 PH martensitic stainless steel powder encapsulated in a multi-component polymer binder. The cylindrical specimens of the nominal dimensions of Ø16 mm and length of 30 mm were prepared in the XY direction. The process parameters were optimized with a layer height of 125 µm and a constant printing velocity of 25 cm^3^/h for structural integrity and deposition stability. All specimens were printed with an oversize pre-defined to compensate for the isotropic volumetric shrinkage occurring during the following thermal consolidation.

After the additive stage, the specimens in the “green” state were subjected to a solvent debinding step in a Wash-1 fluid unit (Markforged Inc., Waltham, MA, USA) using Opteon SF79 for 24 h. This stage allowed the controlled leaching of the primary binder, generating an interconnected porous network (the “brown” state) which is necessary for the integrity of the component during the last thermal stages.

The last consolidation of metallurgical material was carried out in a Sinter-2 tube furnace (Markforged Inc., Waltham, MA, USA) under a controlled reducing atmosphere consisting of 97% Ar/3% H2 forming gas at a constant flow rate of 5 L/min with a controlled dew point of −40 °C during a total 17 h cycle. The thermal profile consisted of a 4 h gradual ramp up for the thermal decomposition of residual binders and an isothermal sintering hold at a peak temperature of 1315 °C for 3 h. At this stage the final densification was obtained by grain boundary diffusion mechanisms. The cycle was finished with a 10-h controlled cooling. All the specimens were then tested in the “as-built” condition without any further mechanical post-processing or surface treatment, in order to evaluate the real effect of the ADAM process on the material properties.

### 2.2. Residual Stress Measurement

Residual stress measurements were conducted directly on the as-sintered outer surface of a single cylindrical specimen, without any prior mechanical grinding, electropolishing, or chemical etching, using a Proto iXRD system (Proto Manufacturing Ltd., LaSalle, ON, Canada) equipped with an MG40 unit. A Mn-Kα X-ray source was employed at a tube voltage of 20 kV and a current of 4 mA. All measurements were performed using a 1 mm diameter collimator, corresponding to an irradiated spot area of approximately 1 mm^2^. The data were collected using the sin^2^ψ method with tilt angles ranging from +39° to −39°. A beta-oscillation of 3.0° was applied to improve grain statistics.

The diffraction patterns were recorded from the {211} crystallographic plane at a nominal diffraction angle (2θ) of 156.4°, using a Cr filter to improve the peak-to-background ratio. The estimated X-ray penetration depth for the 17-4 PH material was approximately 10 µm. Gain correction was performed using the P/G(s) function, and the peak position was determined from the absolute displacement of the diffraction peak maximum.

The residual stress measurements were performed at 12 measurement locations distributed over the outer cylindrical surface of the specimen. Three axial positions were selected at 5, 10, and 15 mm from the specimen edge. At each axial position, measurements were performed at four circumferential orientations of 0°, 90°, 180°, and 270°. Thus, a total of 12 surface locations were investigated on the same specimen. At each measurement location, residual stresses were determined in two principal directions: the axial direction, parallel to the specimen axis, and the tangential direction, perpendicular to the specimen axis (hoop stress).

The standard deviation values were obtained directly from the Proto iXRD system for each individual measurement. These values represent the measurement-related statistical uncertainty associated with the individual X-ray diffraction measurement and should not be interpreted as standard deviations between repeated specimens or as sample-to-sample variability. No replicate specimens were used for the residual stress measurements; therefore, the reported values describe the local measurement uncertainty at each investigated position rather than the reproducibility of the residual stress state across multiple specimens.

### 2.3. Metallographic Sample Preparation

The as-sintered cylindrical specimens were sectioned transversely and longitudinally using a precision diamond cutter with continuous liquid cooling for microstructural evaluation, porosity analysis, and energy-dispersive X-ray spectroscopy (EDS). The transverse section was taken perpendicular to the specimen/build direction, while the longitudinal section was taken parallel to the specimen/build direction.

The cut samples were hot-mounted in a conductive phenolic resin to provide stable and electrically conductive surfaces for microscopic examination.

Metallographic preparation was carried out by successive grinding and polishing. The specimens were ground using water-cooled silicon carbide (SiC) abrasive papers with grit sizes of P240, P400, P800, P1200, and P2500. Final polishing was performed using a 3 µm diamond suspension followed by a 1 µm diamond paste on a synthetic polishing cloth. After each preparation step, the specimens were thoroughly cleaned with ethanol to remove residual abrasive particles.

For porosity evaluation, the polished surfaces were examined in the unetched condition. No chemical etching was applied to these surfaces in order to preserve the original pore morphology and enable reliable identification and evaluation of the pores.

For SEM and EDS analysis, the polished surfaces were subsequently chemically etched using Vilella’s reagent, consisting of 1 g picric acid, 5 mL hydrochloric acid, and 100 mL ethanol. The etching was performed at room temperature for approximately 10–15 s. The etching treatment was used to reveal the microstructural features of the 17-4 PH material and facilitate SEM observation and EDS analysis.

### 2.4. Microstructural and Porosity Characterization

The microstructural characterization of the 17-4PH stainless steel specimen produced by ADAM was carried out using a high-resolution field-emission scanning electron microscope (FE-SEM) ZEISS SUPRA 25 (Carl Zeiss GmbH, Oberkochen, Germany). The microscope is equipped with a Gemini field-emission column that enables high-resolution imaging at low accelerating voltages. The electron source was a field-emission gun (FEG). The sintered material was examined at an accelerating voltage of 20 kV. Micrographs were primarily acquired using the Everhart–Thornley secondary electron (SE2) detector to characterize the surface morphology and microstructural features. The local chemical composition, spatial distribution of alloying elements, and elemental mapping were analyzed by energy-dispersive X-ray spectroscopy (EDS) using an EDAX system (AMETEK Inc., Mahwah, NJ, USA) attached to the microscope.

The obtained SEM micrographs were also used for quantitative porosity analysis using digital image processing in ImageJ version 1.54g. The analysis was performed on two observation planes from the sintered specimen: one transverse section and one longitudinal section. One representative SEM micrograph was evaluated for each orientation at a magnification of 1000×. The same image-processing procedure was applied to both micrographs. Spatial calibration was performed using a reference scale bar of 20 µm corresponding to 94 pixels. Pores were segmented from the solid matrix using global thresholding to determine the two-dimensional porosity area fraction (A_A_). A circularity filter with threshold limits between 0.35 and 1.00 was applied to reduce the influence of microstructural features that could otherwise be incorrectly identified as pores. The “Exclude on edges” function was used to exclude objects intersecting the image boundaries from the quantitative analysis.

Because only one representative SEM micrograph was analyzed for each orientation, the obtained porosity area fractions represent local measurements of the selected transverse and longitudinal observation planes rather than statistical averages. Therefore, no standard deviation was calculated for the image-based porosity measurements. The quantitative results were used to compare the local porosity distribution between the two investigated orientations.

## 3. Results

### 3.1. Microstructural and Chemical Characterization by SEM–EDS

**Figure 1:**
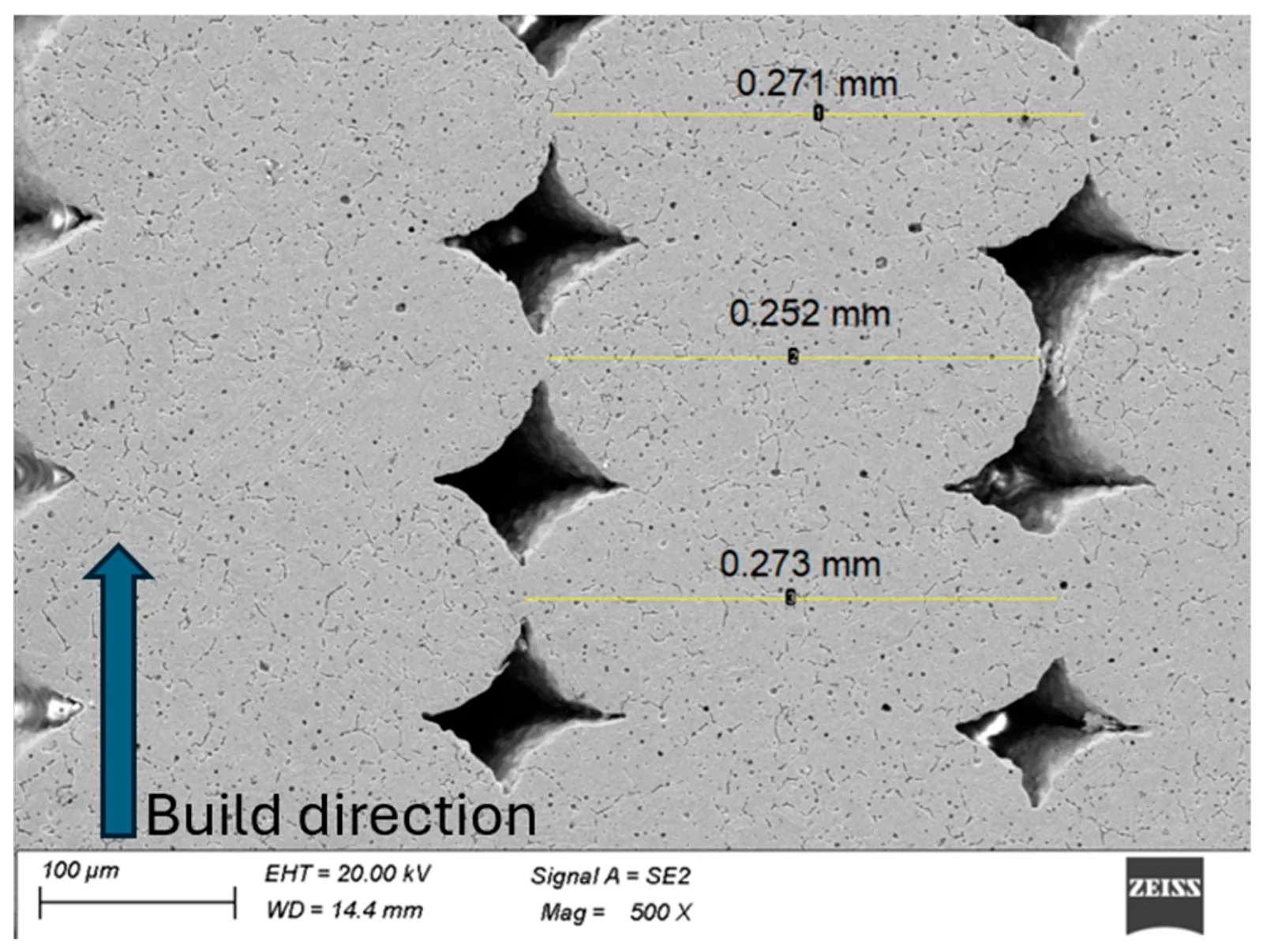
Cross-sectional SEM micrograph of the sintered ADAM-fabricated 17-4PH stainless steel specimen.

Figure 1 shows the cross-sectional SEM micrograph of the ADAM-fabricated 17-4PH stainless steel specimen. The section plane is oriented strictly perpendicular to the direction of material deposition, which allows us to accurately evaluate the contour track geometry and the distribution of macro-defects. Characteristic star-shaped interstitial voids at the junctions of the extruded tracks are clearly observed in a periodic pattern. These systematic gaps are caused by imperfect geometric overlap between neighboring tracks and layers during the stage of filament extrusion, prior to final consolidation at high temperature.

The printing stability was quantitatively evaluated by measuring the width of the inner contour track at three reference locations (indicated by yellow lines in Figure 1). The measurements are highly dimensionally consistent with values from 0.252 mm to 0.273 mm and a mean value of 0.265 mm (standard deviation ≈ 0.012 mm). The localized thinning at position 2 (0.252 mm) is due to small variations in the extrusion head pressure or micro-distortions during the thermal debinding and the subsequent isotropic shrinkage during sintering. In contrast to the pronounced interstitial voids, the bulk core of the individual contours exhibits only fine, homogeneously distributed residual microporosity.

**Figure 2:**
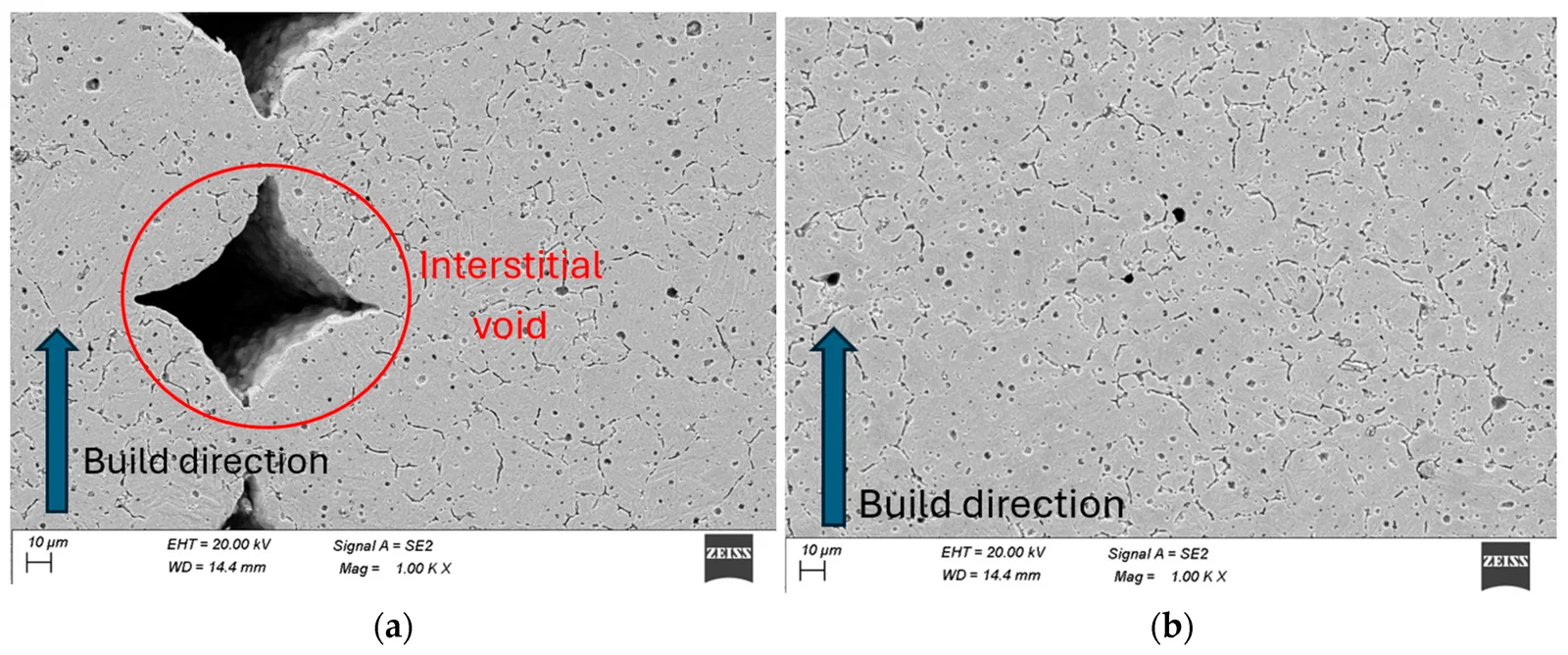
SEM micrographs of the sintered sample microstructure: (**a**) inter-particle/inter-layer porosity, (**b**) grain boundary network with dispersed microporosity.

The SEM micrographs at 1000× magnification show the complex internal structure of the sintered ADAM tracks (Figure 2). The microstructure exhibits two specific features: residual microporosity and intergranular networks.

The transition zones between the extruded layers are clearly visible (Figure 2a). Atomic diffusion has clearly bridged (necked) the previously isolated powder particles, but localized micro-cracks or un-sintered areas near the inter-track junctions suggest the thermal profile may need further optimization to achieve maximum structural integrity. In addition, the high-resolution images reveal a stochastic distribution of fine spherical and sub-spherical micropores within the tracks (Figure 2b). These are the remnants of incomplete densification typical of solid-state sintering. While the large interstitial voids dominate global mechanical behavior, the localized microporosity controls the effective cross-sectional area and intrinsic material density.

Furthermore, grain boundaries are clearly visible at this magnification without the need for chemical etching (Figure 2b), resulting in a predominantly equiaxed grain structure due to the “thermal etching” effect. Dark, elongated features along the grain boundaries indicate either residual binder carbonization products or the formation of secondary phases (e.g., chromium carbides) that may reduce the intergranular corrosion resistance of the stainless steel.

**Figure 3:**
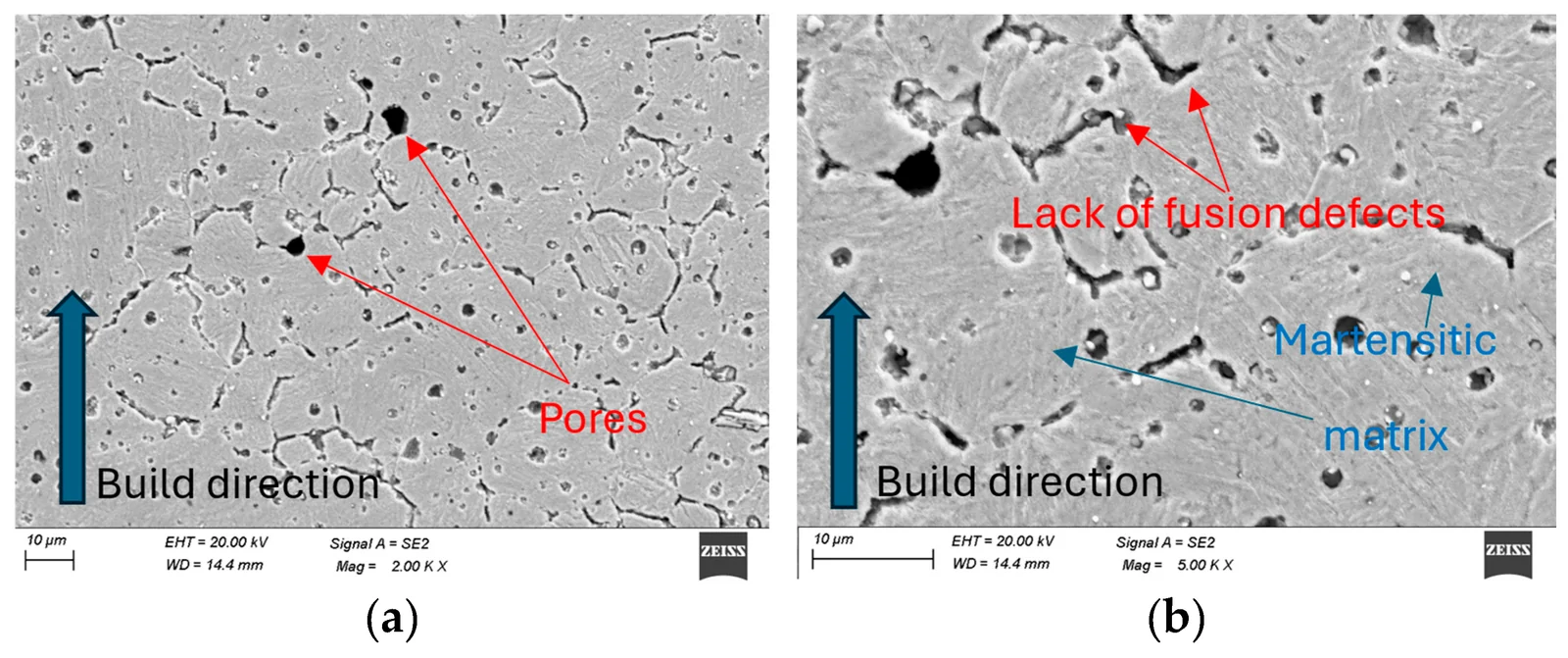
SEM micrographs of ADAM-fabricated 17-4PH steel (transverse section): (**a**) inter-track delamination slit at 2000×; (**b**) martensitic matrix and prior particle boundaries (PPBs) at 5000×.

The microstructural evolution of the 17-4PH stainless steel fabricated by the ADAM process after chemical etching using Vilella’s reagent is shown in Figure 3. The material was examined at magnifications of 2000× and 5000× to evaluate the morphology of the sintered matrix, prior particle boundaries (PPBs), and localized defect regions.

At 2000× magnification (Figure 3a), the microstructure is characterized by a network of prior particle boundaries and dispersed pores. The polygonal morphology reflects the geometry of the original powder particles, while the presence of sintering necks at the particle junctions indicates substantial solid-state mass transport during the sintering cycle. The internal regions of the former powder particles exhibit a fine lath-like morphology. Based on the observed morphology and the processing conditions, the matrix is consistent with a predominantly martensitic microstructure formed during cooling from the sintering temperature. However, the present SEM observations do not provide sufficient information to unambiguously identify all constituent phases or to determine their quantitative phase fractions.

The higher-magnification image (Figure 3b) reveals localized secondary-phase-like features and chemical heterogeneity in the vicinity of PPBs and defect regions. The EDS measurements showed local enrichment in Cr and Nb, accompanied by elevated C concentrations. These observations may be associated with Cr- and Nb-rich secondary phases, potentially including carbide-containing regions. Nevertheless, the crystallographic nature and exact phase composition of these features cannot be conclusively determined from secondary-electron SEM morphology and EDS analysis alone. In particular, the present characterization does not allow unambiguous differentiation between individual carbide types or reliable identification and quantification of possible retained ferritic phases. Phase-specific techniques such as EBSD, X-ray diffraction phase analysis, or transmission electron microscopy would be required for definitive phase identification and quantitative determination of phase fractions.

**Figure 4:**
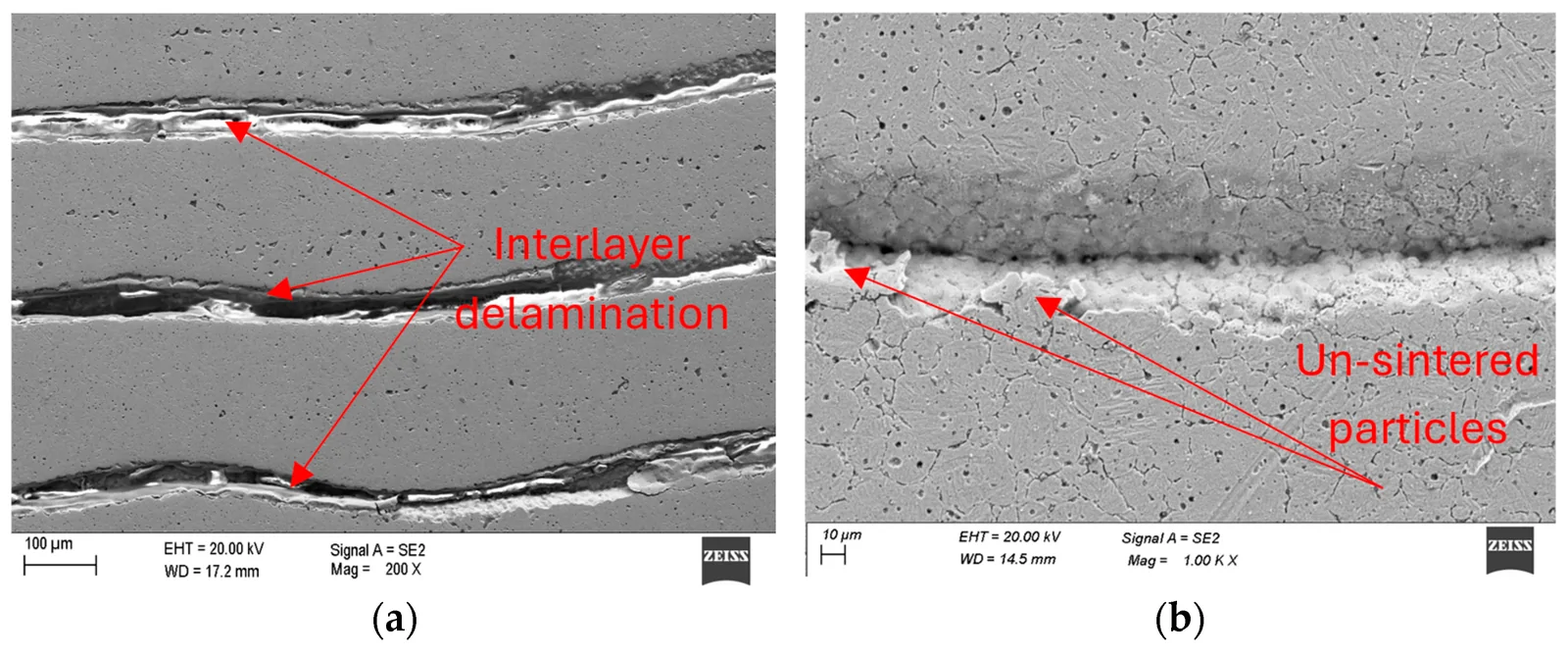
SEM micrographs of ADAM-fabricated 17-4PH steel (longitudinal section): (**a**) continuous inter-layer delamination defects at 200×; (**b**) details of the un-sintered boundary morphology within the delamination zone at 1000×. The build direction is perpendicular to the image plane.

The longitudinal cross-section (build plane) shows a clear stratified architecture with parallel deposition tracks (Figure 4). The most notable observed feature is the presence of elongated interfacial voids or “channel-like” porosity along the direction of deposition (Figure 4a). These discontinuities are localized lack of fusion between successive layers and act as detrimental paths for crack propagation.

The micrograph shows that the interior of the tracks is relatively densely populated with fine sintering micropores, but the overall structural integrity is compromised by inter-laminar delamination (Figure 4b). The intermittent nature of the metallurgical bonding across the layer boundaries implies that the mechanical properties will be highly anisotropic. Such linear defects are typical of the ADAM process, and represent a clear difference to Binder Jetting where the absence of extrusion-based deposition results in a more stochastically distributed porosity, without such pronounced directionally specific planes of weakness.

**Figure 5:**
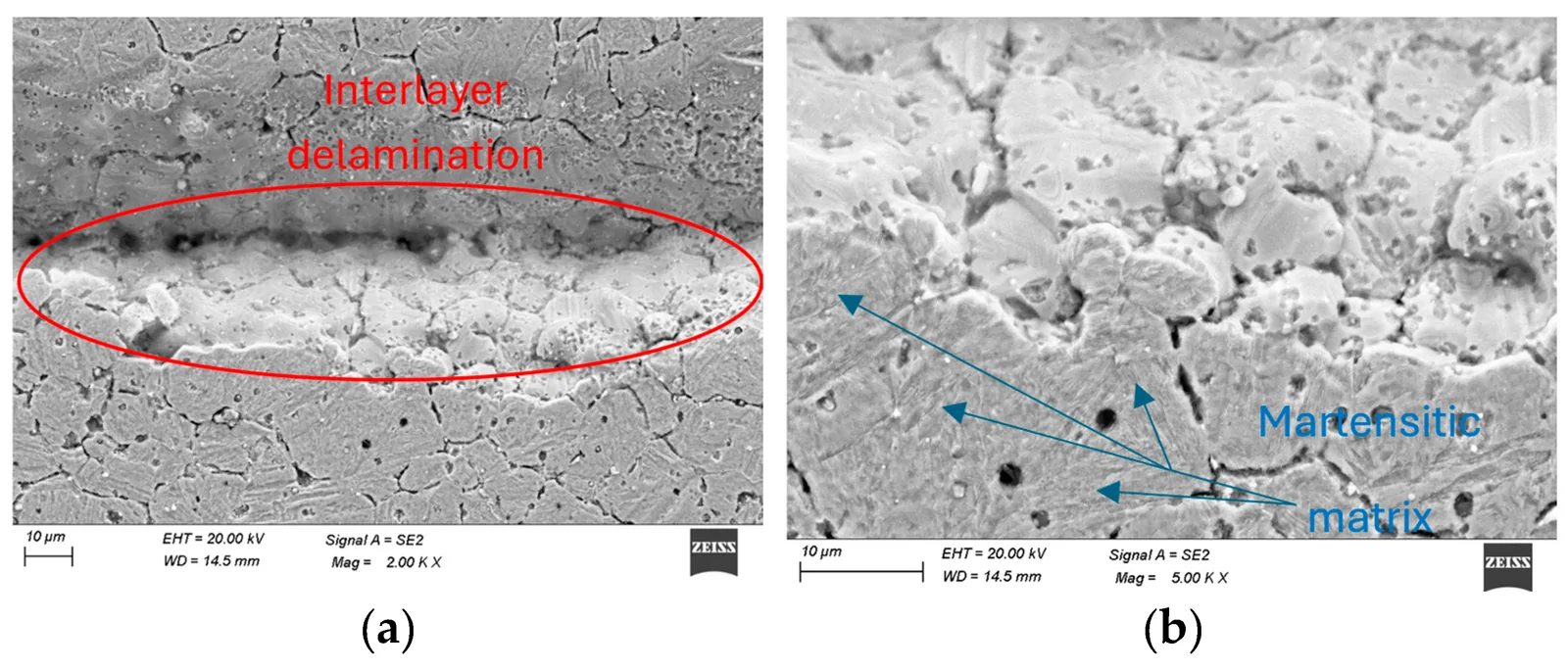
High-magnification SEM micrographs of the delamination zone (longitudinal section): (**a**) morphology of the inter-layer gap at 2000×; (**b**) details of un-sintered spherical powder particles and initial neck formation at 5000×. The build direction is perpendicular to the image plane.

The microstructural characterization at 2000× magnification (Figure 5a) shows the typical features of the material, enhanced by chemical etching with Vilella’s reagent. At this scale a highly densified matrix dominated by the fine needle-like morphology of tempered lath martensite is clearly visible. This phase condition confirms that the material was successfully fully transformed during the cooling from the sintering plateau. The internal architecture is characterized by a well-defined network of prior particle boundaries (PPBs) where solid-state mass transport and diffusion neck formation took place. However, approaching the interfaces where the individual extruded tracks were joined together during printing, a clear gradient in defect distribution is observed together with a strong buildup of fine, spherical micropores (1 to 3 µm).

At ultra-high magnification (5000×, Figure 5b) the nanoscale phenomena occurring at these inter-track junctions are clearly resolved, revealing the direct structural limitations of the ADAM process. At this scale the linear boundaries do not look like homogeneous metallic contacts but rather critical zones of discontinuity. On the one hand, the interface shows long delamination slits which are partially closed, where the adjacent tracks are in mechanical contact, but the sintering kinetics were not enough to fully remove the printing boundary. On the other hand this boundary opens locally, giving rise to large macro-voids with irregular geometric shapes which are mainly located at the triple junctions of the extruded layers. These voids are the result of the limited volumetric flow of the highly viscous polymer–metal filament that could not be filled or closed during the solid-state diffusion step.

The 1–3 µm spherical micropores cluster intensively in the immediate vicinity of both inter-track defect types (Figure 5b), which is a clear physical indicator of the binder decomposition pathway. In the thermal debinding and early sintering stages the gaseous products of the multi-component polymer system preferentially migrate through these inter-layer capillary channels. However, these degradation gases became structurally entrapped and formed localized micro-void fields due to premature neck formation and sealing of outer diffusion pathways during the final sintering plateau. The bulk interior of the sintered tracks shows a high structural integrity, while the co-existence of inter-track slits, triple-junction macro-voids and residual binder-induced microporosity confirms that the layer bonding boundaries are still the most critical structural weak points, directly governing the mechanical anisotropy and the localized stress concentration in ADAM-fabricated components.

**Figure 6:**
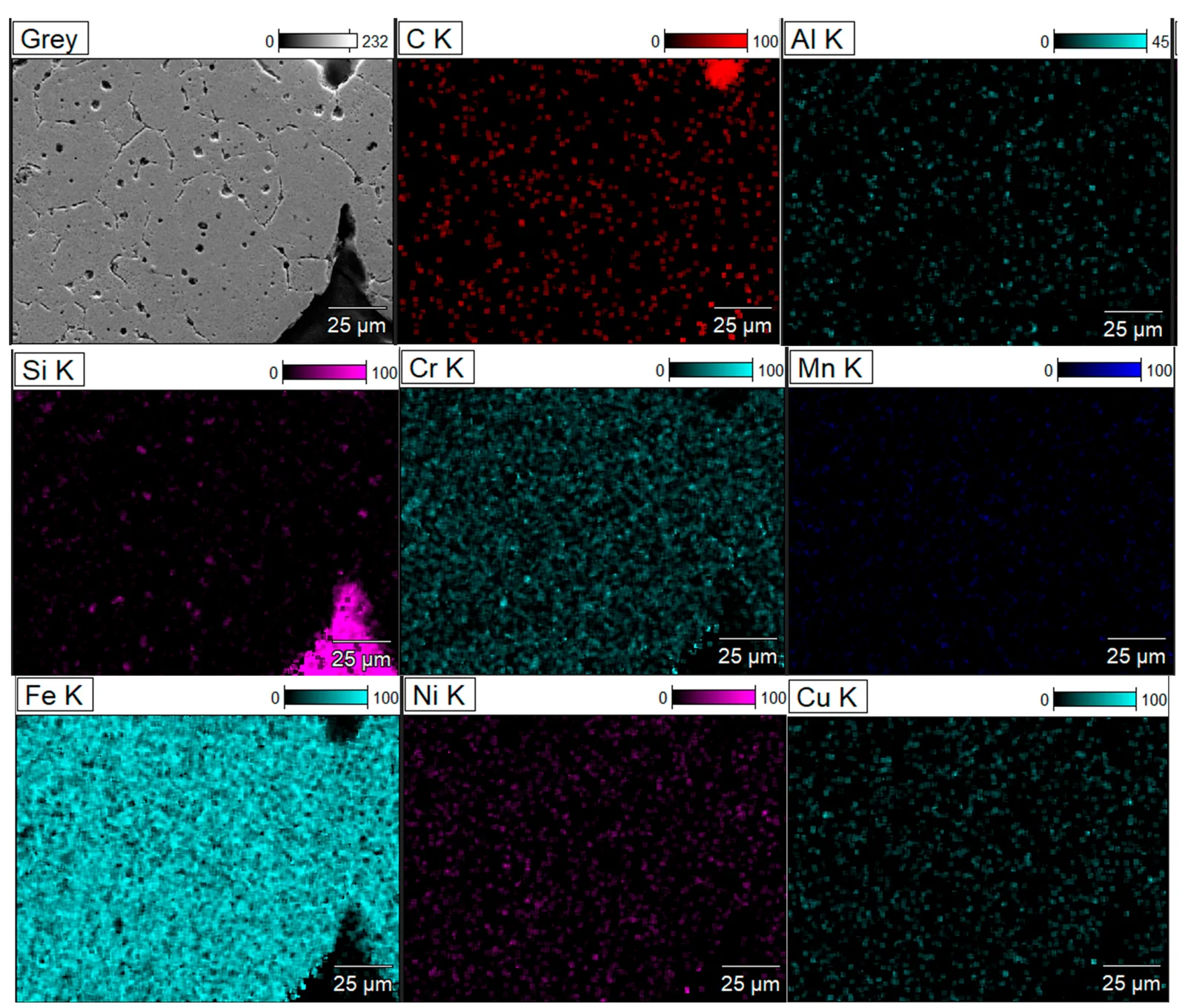
EDS elemental mapping of the ADAM-fabricated 17-4PH steel.

The chemical purity of the sintered 17-4 PH stainless steel matrix and the distribution of alloying elements were characterized through energy-dispersive X-ray spectroscopy (EDS) mapping (Figure 6). Spectroscopic results confirmed the presence of the primary matrix constituents, Iron (Fe), Chromium (Cr) and Nickel (Ni), with a high degree of spatial homogeneity across the sintered tracks. This suggests that the metallic powder was successfully diffused and metallurgically bonded during the sintering cycle. Moreover, the homogenous distribution of Copper (Cu) in the matrix is a key finding, indicating the successful dissolution of Cu during sintering which is a prerequisite for the subsequent precipitation hardening mechanisms normally observed in this alloy grade.

Despite the general stability of the metallic phase, high-resolution mapping revealed localized chemical anomalies related to the light elements and impurities. The Carbon (C K) map shows increased signal intensities, especially in the macro-pore structures and along the grain boundaries (Figure 6, map C K). Interpretation of these data should be made with due regard to surface topography and measurement geometry. The high carbon signal in the voids can be partly explained by the so-called “shadowing” topographic effect or the presence of residual mounting resin in the pores, which was introduced during the metallographic sample preparation. Nevertheless, detection of carbon clusters in dense, non-topographic areas and their correlation with intergranular interfaces point to a real chemical heterogeneity. This phenomenon is most likely caused by the carbonization of the residual polymer binder which was not completely removed during the thermal de-binding step of the ADAM process.

Likewise, localized enrichments of Silicon (Si) and Aluminum (Al) were observed in selected pore structures (Figure 6, maps Si K and Al K). While these signals can be indicative of the presence of oxide inclusions or impurities trapped during the filament extrusion process, the potential contamination from grinding media (e.g., SiC particles) embedded in the porous structure during mechanical polishing cannot be completely excluded. The observed chemical heterogeneity, together with the channel-like porosity already identified, is a crucial factor that may cause localized corrosion. These findings underscore the critical need for rigorous control of surface purity and optimization of the thermal processing cycle to remove harmful chemical phases and maintain the performance integrity of ADAM-fabricated 17-4 PH stainless steel.

### 3.2. Quantitative Chemical Analysis via Point EDS

**Figure 7:**
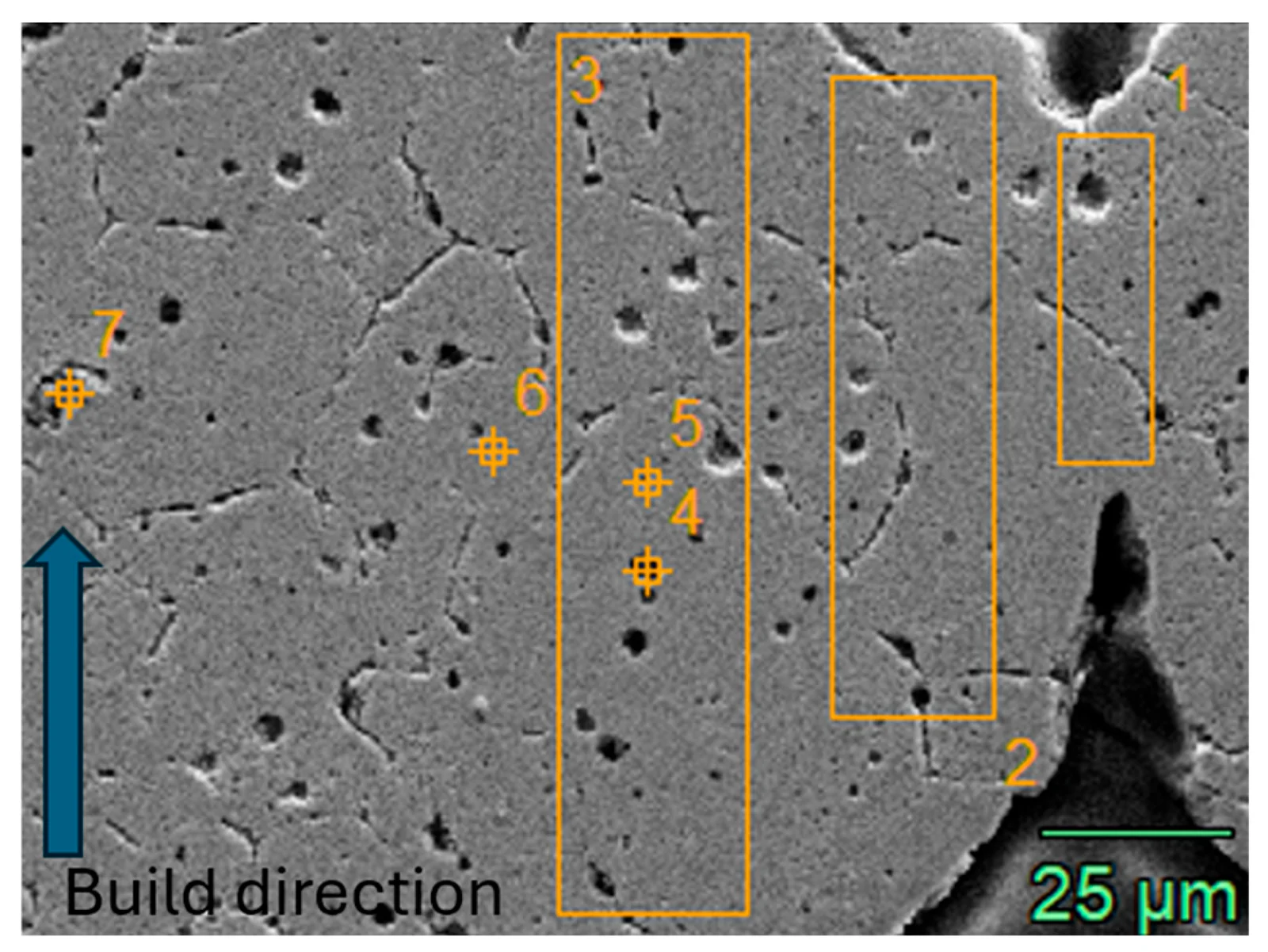
SEM micrograph showing the locations of EDS points.

The quantitative point analysis data (Table 2) corroborates and complements the elemental mapping results. The SEM micrograph in Figure 7 illustrates the locations of the EDS measurement points within the dense matrix and heterogeneous regions of the ADAM-fabricated 17-4 PH stainless steel.

Matrix Chemical Stability. Bulk material is represented by points 1, 2, 3, 5, and 6 (Figure 7). The concentrations of Chromium (Cr: 16.5–17.2 wt.%) and Nickel (Ni: 3.1–3.7 wt.%) values fit exactly the nominal 17-4 PH specifications, as shown in Table 2. The constant content of Copper (Cu: ~4 wt.%, Table 2) proves that the hardening elements are evenly distributed in the solid solution, which has the potential for subsequent aging treatments.

Inclusions of Anomalies and Impurities (Points 4 and 7): Large stoichiometric deviations are quantitatively summarized in Table 2 and spatially designated in Figure 7.

Niobium (Nb) and Chromium (Cr) Segregation: As shown in Point 7 of Table 2, a strong enrichment of Niobium (16.31 wt.%) and Chromium (42.58 wt.%) is detected. This indicates the formation of complex intermetallic phases or Niobium-rich carbides (NbCs) which preferentially precipitate at low-density sites or previous particle boundaries.

Silicon and Impurity Considerations: The silicon contents at Point 4 and Point 7 are remarkably elevated (Table 2). Rather than being intrinsic bulk constituents, these anomalies are primarily attributed to microscopic residues of the ceramic release layer—a multi-material interface utilized in the ADAM process to separate the part from the build raft—which remained mechanically trapped within the open pore network and channel-like structures despite ultrasonic cleaning. Additionally, potential contributions from metallographic preparation (such as abrasive particles embedded in pores) cannot be entirely excluded.

Furthermore, regarding the apparent carbon enrichment (e.g., 1.61 wt.% at Point 7), we acknowledge that standard EDS lacks absolute quantitative accuracy for light elements due to low X-ray yields and potential hydrocarbon vacuum contamination; thus, carbon distributions are interpreted strictly as qualitative indicators, keeping in mind possible topographical shadow effects and residual mounting resin from preparation, as described in Section 2.3.

Quantitative analysis of Point 7 (Table 2) shows an elevated carbon concentration of 1.61 wt.% (6.67 at.%). Compared to the matrix average (~1.1 wt.%), this indicates localized carbon enrichment in the vicinity of the structural defects, potentially associated with residual binder-derived species.

### 3.3. Residual Stress Analysis

**Figure 8:**
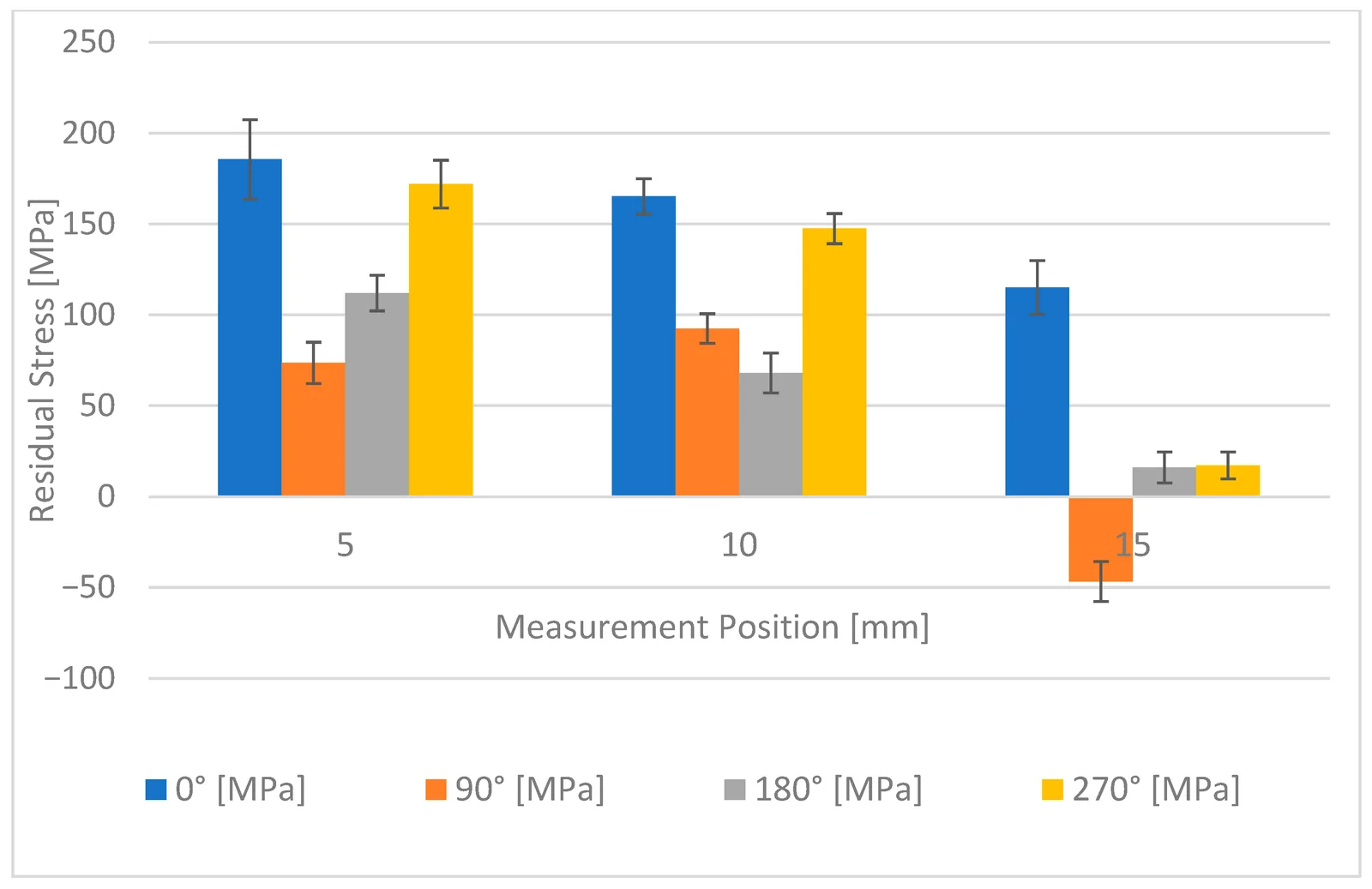
Residual stress distribution in the axial direction at different measurement positions.

The measured residual stress distribution on the outer surface of the investigated 17-4PH specimen showed a clear spatial variation along the specimen axis (Figure 8, Table 3). The highest tensile axial stress of 185.6 ± 21.9 MPa was measured at the 5 mm axial position and 0° circumferential orientation. At the same circumferential orientation, the axial stress decreased to 165.3 ± 9.7 MPa at 10 mm and 115.1 ± 14.8 MPa at 15 mm. At the 90° circumferential orientation, the axial stress decreased from 73.6 ± 11.4 MPa at 5 mm to −46.7 ± 10.9 MPa at 15 mm, indicating a local transition from tensile to compressive stress along the measured surface.

These results demonstrate a spatially non-uniform residual stress state over the investigated outer surface. The axial component was generally tensile, with the highest values measured at the 5 mm position, while locally compressive stress was detected at the 15 mm position at 90° circumferential orientation. The observed spatial variation may be related to non-uniform thermal contraction and stress redistribution during cooling from the sintering temperature. However, because the measurements were performed only at discrete positions on the outer surface of a single specimen, the results should be interpreted as a spatial characterization of the investigated surface rather than as evidence of a through-thickness or peripheral-to-core residual stress gradient.

All measured axial residual stress values remained below the yield strength (Rp0.2) reported for 17-4 PH stainless steel. Therefore, the measured residual stress magnitudes indicate that the investigated as-sintered specimen was not subjected to residual stresses approaching the elastic limit.

**Figure 9:**
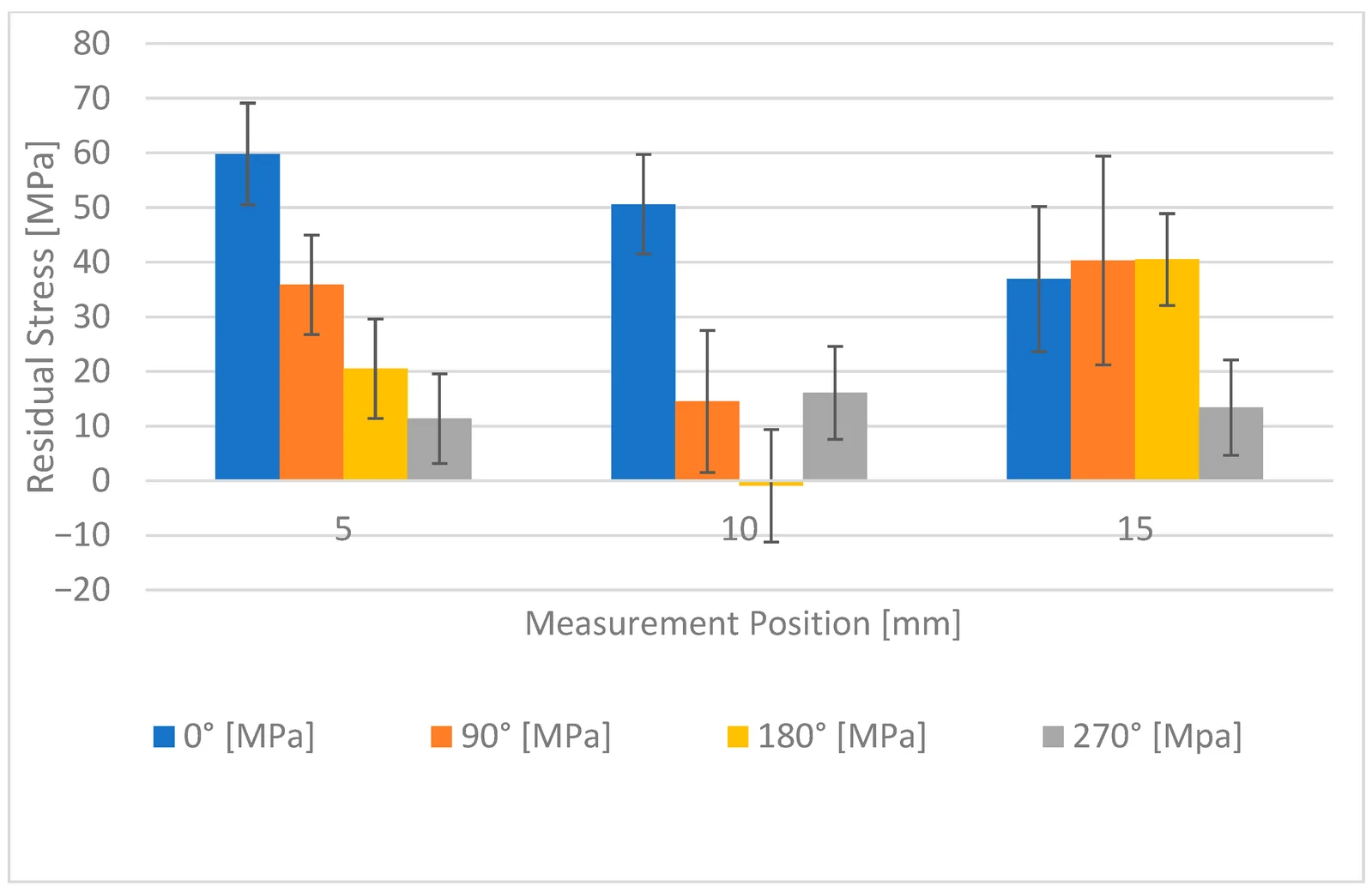
Residual stress distribution in the tangential direction at different measurement positions.

The distribution of the tangential (hoop) residual stress components is generally lower in magnitude than that of the corresponding axial stresses (Figure 9, Table 4). The maximum measured tangential stress was 59.8 ± 9.3 MPa at the 0° orientation and 5 mm position. This value was substantially lower than the maximum measured axial stress at the corresponding location. The measured results indicate that, for the investigated specimen, the residual stress state was more pronounced in the axial direction than in the tangential direction.

At the investigated axial positions, several tangential stress values were close to zero, including −0.9 ± 10.3 MPa at the 180° circumferential orientation and 10 mm axial position. Based on the measured values, no pronounced tangential residual stress concentrations were observed at the investigated locations on the outer surface of the specimen.

### 3.4. Porosity Evaluation

The quantitative porosity analysis was performed on two SEM observation planes from the sintered specimen: one transverse section and one longitudinal section. One representative SEM micrograph was analyzed for each orientation. The same image-processing procedure was applied to both images using ImageJ software, including grayscale thresholding and particle filtering based on circularity. The analyzed images were used to determine the number of detected pores, their cumulative area, and the corresponding two-dimensional porosity area fraction (A_A_). Since only one representative image was analyzed for each orientation, the reported values represent local measurements of the selected observation planes and are not statistical averages. Consequently, standard deviations were not calculated for the image-based porosity measurements.

**Figure 10:**
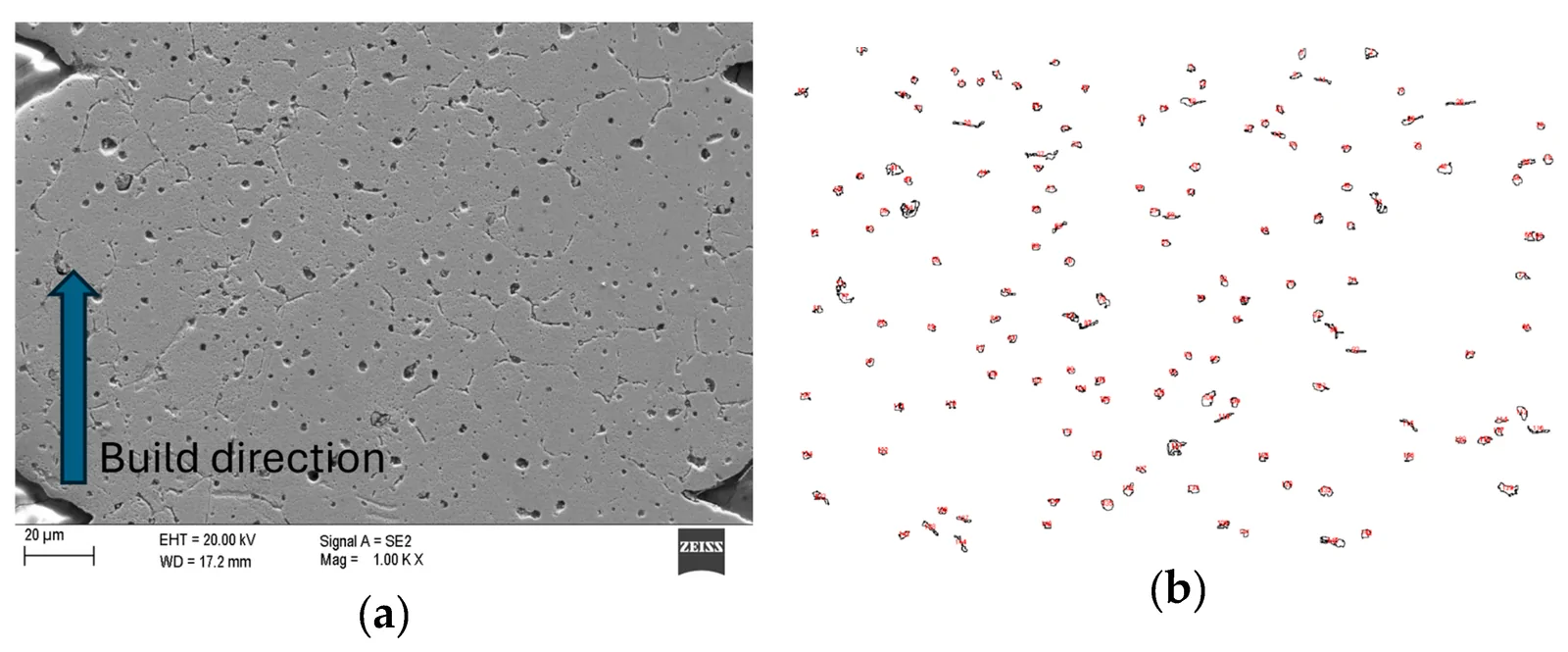
Quantitative porosity analysis in the transverse section: (**a**) original SEM micrograph of the sintered matrix; (**b**) thresholded binary image illustrating the segmented micro-voids.

The quantitative analysis of the transverse section (Figure 10) revealed a relatively consolidated matrix with a limited number of visible pores. A total of 145 pores were identified within the analyzed region, with a cumulative pore area of 493.19 µm^2^. The calculated porosity area fraction (A_A_) was 1.45% (Figure 10). The observed pores were predominantly small and relatively uniformly distributed within the analyzed field.

**Figure 11:**
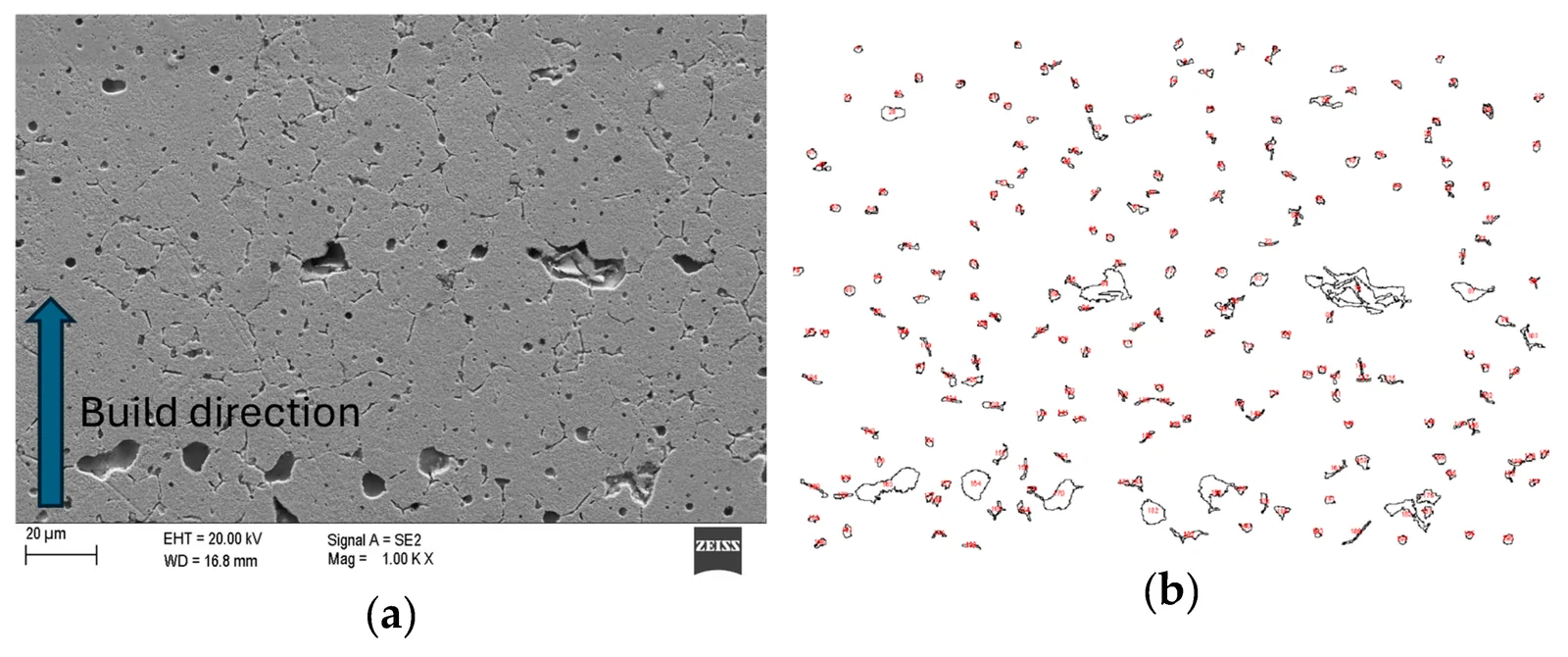
Quantitative porosity analysis in the longitudinal section: (**a**) original SEM micrograph showing elongated inter-layer defects; (**b**) thresholded binary image illustrating the segmented micro- and macro-voids.

In the longitudinal section (Figure 11), analyzed using the same image-processing procedure, a higher density of voids was observed. The image analysis identified 199 discrete pores with a cumulative pore area of 1214.09 µm^2^, corresponding to a porosity area fraction (A_A_) of 3.48% (Figure 11). Compared with the transverse section, the longitudinal section therefore exhibited a higher local porosity area fraction. Several elongated voids and interlayer-related defects were observed in this section, indicating a directional dependence of the observed pore distribution associated with the layer-wise manufacturing structure.

The quantitative porosity analysis was based on two-dimensional SEM image analysis performed using ImageJ. The measured porosity area fractions were 1.45% in the transverse section and 3.48% in the longitudinal section. The higher porosity area fraction observed in the longitudinal section is associated with elongated, channel-like inter-layer defects oriented along the deposition direction. These results indicate pronounced anisotropy in the spatial distribution of porosity. It should be noted that the reported values represent local two-dimensional area fractions obtained from the selected SEM observation planes and therefore should not be interpreted as direct measurements of the overall three-dimensional volumetric porosity of the specimen. The results are intended primarily to characterize the directional distribution and morphology of porosity within the investigated material.

## 4. Discussion

The experimental results of this study provide key insights into the complex correlations between extrusion-based processing, microstructural evolution, chemical homogeneity, porosity distribution, and the resulting residual stress state in 17-4PH stainless steel manufactured via Atomic Diffusion Additive Manufacturing (ADAM). Macroscopic and microscopic structural characterizations highlighted a highly anisotropic distribution of porosity, which is a characteristic feature of indirect, extrusion-based metal additive manufacturing (MEX) technologies. The transverse cross-section showed a high degree of consolidation with a relatively low area fraction of porosity (AA = 1.45%) and fine, stochastically distributed micropores within the bulk of the deposited tracks. In contrast, the longitudinal section exhibited a considerably higher porosity area fraction (AA = 3.48%). The observed microstructural anisotropy and the typical lack-of-fusion defects are consistent with the mechanisms described by Henry et al. [24] and Galati et al. [25]. The measured porosity values and the directional morphology of the observed defects are also in good agreement with the results reported by Singh et al. [49] and Mondésir et al. [50]. These authors confirmed that, when composite filaments are used in indirect extrusion-based processes, elongated directional voids can occur at layer boundaries and may not be completely eliminated during conventional ambient-pressure sintering.

Furthermore, the periodic star-shaped interstitial voids observed at the junctions of the extruded tracks in the transverse plane can be attributed to incomplete geometrical overlap between neighboring deposited beads during the extrusion process. The pronounced directional porosity and interlayer delamination may act as local stress concentrators and may provide preferential locations for crack initiation, particularly under cyclic loading. Their influence on mechanical performance is expected to depend not only on the total porosity fraction, but also on pore morphology, size, orientation, and spatial distribution. Consequently, the higher porosity observed in the longitudinal section may be particularly relevant for loading conditions in which the applied stress is oriented relative to the elongated interlayer defects. These defects may promote local stress concentration and facilitate crack initiation and propagation under cyclic or complex loading conditions. This behavior differs from alternative powder metallurgy-based methods, such as Binder Jetting (BJ) [51,52], where the absence of continuous linear extrusion tracks and the use of a powder bed generally results in a more stochastically distributed and less directionally organized porosity network.

The high-temperature thermal profile, including a 3 h holding period at 1315 °C, promoted grain-boundary diffusion and sintering between the previously discrete powder particles, resulting in a predominantly consolidated microstructure with an equiaxed martensitic morphology. However, the presence of localized micro-cracks and non-sintered regions close to the inter-track junctions indicates that the sintering process did not completely eliminate the geometrical features inherited from the green state. This suggests that the final densification and defect population are influenced by both the initial deposition geometry and the subsequent sintering kinetics. Localized chemical anomalies identified by EDS mapping and point analyses further indicate that the chemical state of the material may be affected by the preceding binder removal process.

While the principal matrix elements (Fe, Cr, Ni) and the precipitation-hardening element (Cu) were found to be relatively homogeneous within the investigated bulk tracks, localized compositional deviations were observed within macro-voids and at prior particle boundaries. The high carbon concentration, reaching up to 1.61 wt.% at Point 7, suggests the possible presence of carbon-rich residues associated with incomplete thermal decomposition of the multi-component polymer binder during the debinding stage. Romero et al. [34] and Opoz et al. [45] reported similar chemical anomalies and early precipitation of secondary phases related to the degradation of residual binder in extrusion-processed materials. The degradation and carbonization of the polymer matrix have also been described by Pompeo [53] and Lieberwirth et al. [54], indicating that the efficiency of binder removal can influence the final chemical condition and metallurgical purity of sintered steels.

The localized carbon enrichment observed in the investigated regions was accompanied by high concentrations of chromium (42.58 wt.%) and niobium (16.31 wt.%). These local compositional deviations may be associated with Cr- and Nb-rich secondary phases or chemically enriched regions at prior particle boundaries. However, the present EDS measurements alone are not sufficient to unambiguously identify the crystallographic nature or specific phase constitution of these regions, including individual carbide phases such as M23C6 or NbC. Definitive identification and quantification of the corresponding phases would require complementary phase-specific characterization, such as EBSD, X-ray diffraction phase analysis, or transmission electron microscopy. The observed local chemical segregation could potentially be associated with chromium redistribution in the vicinity of these regions; however, chromium depletion in the surrounding matrix was not directly quantified in the present study. Therefore, the observed chemical heterogeneity should be interpreted as an indication of local metallurgical variation rather than direct evidence of a specific phase constitution, sensitization mechanism, or corrosion behavior [42].

Moreover, the very high silicon concentration, reaching up to 13.31 wt.% at Point 4, was detected within the porous network. This enrichment may indicate localized trapping of external impurities originating either from feedstock contamination or from SiC-containing abrasive media introduced during metallographic sample preparation [33]. Consequently, the observed silicon concentration should be interpreted cautiously, as the present EDS measurements do not allow the origin of the silicon to be determined unambiguously.

The sin^2^ψ X-ray diffraction measurements performed on the investigated cylindrical specimen revealed a spatially non-uniform residual stress state over the measured outer surface. The highest tensile axial stress was measured at the 5 mm axial position and 0° circumferential orientation, reaching 185.6 ± 21.9 MPa. At the 15 mm axial position and 90° circumferential orientation, a locally compressive axial stress of −46.7 ± 10.9 MPa was measured. The observed spatial variation in residual stress may be associated with non-uniform thermal contraction and stress redistribution during the cooling stage following sintering [9,10]. Since the measurements were performed at discrete locations on a single specimen, the observed stress distribution should be considered representative of the investigated specimen and should not be interpreted as a statistically reproducible trend across multiple specimens.

The measured residual stress state can be related to the thermal history characteristic of the ADAM process. In contrast to fusion-based additive manufacturing processes, such as laser or electron beam powder bed fusion, where rapid and highly localized melting and solidification can generate substantial thermal gradients and residual stresses [53,54], ADAM involves subsequent debinding and high-temperature sintering in a furnace. The prolonged thermal cycle and elevated sintering temperature can reduce stresses generated during the preceding extrusion stage and modify the final residual stress state during furnace cooling [55]. Therefore, the residual stresses measured in the present study should primarily be interpreted in the context of the thermal history associated with extrusion, debinding, sintering, and subsequent cooling.

When comparing the present results with other metal additive manufacturing routes, fusion-based processes such as Laser Powder Bed Fusion (L-PBF) and Directed Energy Deposition (DED) are generally associated with strong localized thermal gradients resulting from repeated melting and rapid solidification. These thermal conditions can lead to substantial residual stresses in as-built components [56,57,58]. Recent investigations of 17-4PH stainless steel processed by L-PBF have reported significant residual stress levels associated with localized heat input and repeated thermal cycling [56,57,58]. In contrast, studies of 17-4PH and closely related precipitation-hardening stainless steels fabricated by DED have demonstrated the importance of localized heat input, process parameters, repeated reheating, and the resulting thermal history on the material response [59,60,61]. In particular, Sharma et al. [59] investigated QT17-4+ steel produced by DED and demonstrated the strong influence of DED process parameters, including laser power, scan speed, powder feed rate, and hatch overlap, on the resulting material density and properties. Yu et al. [60] investigated 17-4PH stainless steel fabricated by laser direct metal deposition and showed that the thermal history associated with laser remelting influenced porosity, microstructural evolution, and mechanical anisotropy. Lyu et al. [61] further demonstrated that the intrinsic thermal cycles associated with subsequent deposition passes affect the microstructure and mechanical properties of 17-4PH steel fabricated by wire-arc DED. In contrast, the ADAM process includes a subsequent high-temperature furnace treatment, which substantially modifies the thermal history of the material before the final component is obtained [40]. Therefore, the maximum axial residual stress measured in the present study should be interpreted in the context of the different thermal histories associated with the respective AM processes. Direct quantitative comparison between ADAM, L-PBF, and DED should nevertheless be made cautiously because residual stress values depend strongly on material condition, component geometry, processing parameters, heat treatment, measurement technique, and measurement location [56,57,58,59,60,61].

The maximum measured axial residual stress of 185.6 ± 21.9 MPa and the maximum tangential stress of 59.8 ± 9.3 MPa remained below the yield strength (Rp0.2) of 17-4PH stainless steel. The substantially higher magnitude of the axial component compared with the tangential component indicates that the residual stress state was more pronounced in the axial direction at the investigated surface locations. However, the measured values represent local surface stresses and do not provide sufficient information to determine the complete three-dimensional residual stress field within the specimen [56].

The measured stress magnitudes indicate that the investigated as-sintered specimen was not subjected to residual stresses approaching the material yield strength. Nevertheless, the presence of directional channel-like porosity and local chemical heterogeneities identified in the microstructural analysis may influence the mechanical response independently of the measured residual stress state. In particular, elongated interlayer defects may locally amplify the effect of applied and residual stresses and may therefore contribute to crack initiation under cyclic loading. Consequently, the residual stress results should be considered together with the observed porosity and microstructural defects when assessing the structural integrity of ADAM-fabricated 17-4PH stainless steel [58]. Although the present study does not directly quantify the stress concentration around individual pores or interlayer defects, the simultaneous presence of directional defects and spatially varying residual stresses indicates that their combined effect should be considered in future mechanical and fatigue investigations.

From an industrial application perspective, the investigated ADAM process shows potential for the production of geometrically complex metal components; however, the observed directional porosity, interlayer defects, and localized chemical heterogeneities should be considered when assessing suitability for demanding service conditions. In addition to these material-related limitations, the relatively low resolution and dimensional accuracy of ADAM compared with higher-resolution additive manufacturing technologies, such as Laser Powder Bed Fusion (L-PBF), may impose limitations on the fabrication of small components and complex geometries containing fine features, narrow channels, thin walls, or sharp transitions. These limitations are related to the extrusion-based deposition mechanism, the dimensions of the deposited tracks, and the subsequent dimensional changes associated with debinding and sintering. Consequently, features with dimensions approaching the characteristic deposition-track size may be difficult to reproduce accurately, while sintering shrinkage may further influence the final dimensions and geometric fidelity of the component. Previous investigations of the ADAM process have demonstrated that dimensional accuracy can be affected by process-related parameters and differs from that achievable by other metal additive manufacturing technologies [26,29]. Therefore, although ADAM can provide advantages for the fabrication of complex metal components, applications requiring high dimensional accuracy and fine geometric resolution may be better suited to processes such as L-PBF [26,29]. Components subjected to high cyclic loads or applications requiring high fatigue and fracture resistance may require additional post-processing and qualification. Hot Isostatic Pressing (HIP) represents a potential post-processing route for reducing internal porosity and improving structural integrity, although its effectiveness for the specific defect population observed in this ADAM-fabricated 17-4PH material would require experimental verification.

Finally, the results were obtained from cylindrical specimens and from a limited number of measurement locations on a single specimen. Therefore, extrapolation to complex geometries, thin-walled components, substantially different cross-sectional dimensions, or other processing conditions should be performed with caution. Geometry, local thermal history, binder removal, sintering shrinkage, wall thickness, surface-to-volume ratio, and track arrangement may influence the resulting microstructure, porosity distribution, dimensional changes, and residual stress state [40,49,50,56,57]. In particular, changes in wall thickness and surface-to-volume ratio may affect the transport of binder decomposition products during debinding and heat transfer during sintering and cooling. Consequently, the results obtained in this study should be considered representative of the investigated cylindrical specimen geometry, material condition, printing parameters, and sintering cycle rather than of ADAM-fabricated components in general.

The observed directional porosity, interlayer defects, chemical heterogeneities, and residual stress gradients indicate the need for further evaluation of their influence on the mechanical performance of ADAM-fabricated 17-4PH stainless steel. Future investigations will therefore focus on the mechanical and, in particular, fatigue behavior of the material, with emphasis on the relationship between defect morphology, porosity orientation, residual stress state, and crack initiation and propagation.

## 5. Conclusions

This study presented a detailed microstructural, chemical, porosity, and residual stress characterization of 17-4PH stainless steel components fabricated by the Atomic Diffusion Additive Manufacturing (ADAM) process. Based on the experimental results, the following conclusions can be drawn.

The ADAM process resulted in pronounced directional porosity anisotropy. The transverse section exhibited a lower porosity area fraction of 1.45%, whereas the longitudinal section showed a higher value of 3.48%, mainly associated with elongated channel-like defects along the build direction. These defects represent important structural discontinuities that may influence the mechanical behavior of the material.

Sintering at 1315 °C promoted solid-state diffusion and the formation of sintering necks between the powder particles, resulting in a predominantly equiaxed microstructural morphology. Nevertheless, incomplete consolidation remained evident in the form of periodic star-shaped inter-track voids and localized microporosity, particularly near the boundaries between extruded tracks.

EDS analysis confirmed a relatively homogeneous distribution of the principal alloying elements Fe, Cr, Ni, and Cu within the dense matrix. In contrast, pronounced local chemical variations were detected within selected macro-voids and defect regions. The measured local concentrations included 1.61 wt.% C, 13.31 wt.% Si, 42.58 wt.% Cr, and 16.31 wt.% Nb. These localized deviations indicate chemical heterogeneity associated with defect regions; however, the EDS results alone do not allow the specific chemical phases or the exact origin of these anomalies to be conclusively identified.

The residual stress measurements revealed a spatially non-uniform stress state over the investigated outer surface of the specimen. The maximum measured tensile stresses were 185.6 ± 21.9 MPa in the axial direction and 59.8 ± 9.3 MPa in the tangential direction. A locally compressive axial stress of −46.7 ± 10.9 MPa was measured at the 15 mm axial position and 90° circumferential orientation. All measured residual stresses remained below the yield strength of 17-4PH stainless steel. Since the measurements were performed at discrete locations on a single specimen, the results should be considered representative of the investigated specimen rather than a statistically reproducible residual stress distribution.

Overall, the results demonstrate that the main structural limitations of the investigated ADAM-fabricated 17-4PH material are associated with directional inter-track porosity and localized chemical heterogeneity rather than high residual stress magnitudes. Future work should therefore focus on optimizing extrusion track overlap and thermal debinding and sintering conditions to reduce directional defects, improve densification, and minimize localized chemical anomalies.

## Figures and Tables

**Table 1:** Chemical composition of 17-4PH stainless steel [48].

	% of Weight								
Element	C	Cr	Ni	Cu	Nb	Mn	Si	P	S
Manufacturer specification	0.07 max	15–17.5	3–5	3–5	0.15–0.45	1.0 max	1.0 max	0.04 max	0.03 max

**Table 2:** Chemical composition (wt.%) of selected micro-regions determined by EDS point analysis.

	C	Al	Si	Cr	Mn	Ni	Cu	Fe
point 1	1.12	0.07	0.69	17.19	0.00	3.11	4.12	73.69
point 2	1.17	0.05	0.55	16.72	0.13	3.41	4.30	73.66
point 3	1.03	0.06	0.73	17.14	0.20	3.31	3.62	73.92
point 4	1.15	0.14	13.31	32.77	3.38	1.36	2.94	38.83
point 5	1.11	0.04	0.21	16.53	0.00	3.74	3.61	74.76
point 6	0.92	0.03	0.19	16.96	0.00	3.18	4.08	74.65
point 7	1.61	0.16	9.53	42.58	0.96	1.40	1.10	25.34

**Table 3:** Residual stress distribution in the axial direction.

Angle [°]	Position [mm]	Axial Stress [MPa]	Measurement Uncertainty [MPa]
0°	5	185.6	21.9
	10	165.3	9.7
	15	115.1	14.8
90°	5	73.6	11.4
	10	92.5	8.1
	15	−46.7	10.9
180°	5	112	9.8
	10	68.1	10.9
	15	16	8.5
270°	5	172	13.2
	10	147.5	8.3
	15	17.2	7.3

**Table 4:** Residual stress distribution in the tangential direction.

Angle [°]	Position [mm]	Tangential Stress [MPa]	Measurement Uncertainty [MPa]
0°	5	59.8	9.3
	10	50.6	9.1
	15	36.9	13.3
90°	5	35.9	9.1
	10	14.5	13
	15	40.3	19.1
180°	5	20.5	9.1
	10	−0.9	10.3
	15	40.5	8.4
270°	5	11.4	8.2
	10	16.1	8.5
	15	13.4	8.7

## Data Availability

The original contributions presented in this study are included in the article. Further inquiries can be directed to the corresponding author.
